# Does SARS-CoV-2 affect neurodegenerative disorders? TLR2, a potential receptor for SARS-CoV-2 in the CNS

**DOI:** 10.1038/s12276-022-00755-7

**Published:** 2022-04-08

**Authors:** Marcell P. Szabo, Michiyo Iba, Avindra Nath, Eliezer Masliah, Changyoun Kim

**Affiliations:** 1 Molecular Neuropathology Section, Laboratory of Neurogenetics, National Institute on Aging, National Institutes of Health, Bethesda, MD 20892 USA; 2 Section of Infections of the Nervous System, National Institute of Neurological Disorders and Stroke, National Institutes of Health, Bethesda, MD 20892 USA; 3 Division of Neuroscience, National Institute on Aging, National Institutes of Health, Bethesda, MD 20892 USA

**Keywords:** Neurodegeneration, Neurodegenerative diseases

## Abstract

The coronavirus (COVID-19) pandemic, caused by severe acute respiratory system coronavirus 2 (SARS-CoV-2), has created significant challenges for scientists seeking to understand the pathogenic mechanisms of SARS-CoV-2 infection and to identify the best therapies for infected patients. Although ACE2 is a known receptor for the virus and has been shown to mediate viral entry into the lungs, accumulating reports highlight the presence of neurological symptoms resulting from infection. As ACE2 expression is low in the central nervous system (CNS), these neurological symptoms are unlikely to be caused by ACE2-virus binding. In this review, we will discuss a proposed interaction between SARS-CoV-2 and Toll-like receptor 2 (TLR2) in the CNS. TLR2 is an innate immune receptor that recognizes exogenous microbial components but has also been shown to interact with multiple viral components, including the envelope (E) protein of SARS-CoV-2. In addition, TLR2 plays an important role in the pathogenesis of neurodegenerative diseases such as Alzheimer’s disease (AD) and Parkinson’s disease (PD). Based on these observations, we hypothesize that TLR2 may play a critical role in the response to SARS-CoV-2 infiltration in the CNS, thereby resulting in the induction or acceleration of AD and PD pathologies in patients.

## SARS-CoV-2: Receptor linked to the neurological toll of COVID-19

Interaction between the SARS-CoV-2 virus and a membrane protein in the central nervous system (CNS) that recognizes foreign substances is implicated in causing some of the neurological symptoms of COVID-19. Toll-like receptor 2 protein (TLR2) binds to components of microbes, including specific parts of the SARS-CoV-2 virus, as part of its role in natural immunity. It is widely present in many brain cells and is known to be involved in the pathogenesis of neurodegenerative diseases including Alzheimer’s and Parkinson’s diseases. Changyoun Kim, Eliezer Masliah and colleagues at the National Institutes of Health in Bethesda, USA, review the emerging evidence suggesting TLR2 plays a critical role in the response to SARS-CoV-2 virus infiltration into the CNS, inducing or accelerating neurological degeneration. Deeper understanding of the link could help develop new treatments for the neurological symptoms of COVID-19.

## Introduction

The coronavirus pandemic, which is caused by severe acute respiratory system coronavirus 2 (SARS-CoV-2), has resulted in the infection of over 326 million people, with more than 5.5 million deaths due to the disease^1^. The disease is considered to be primarily respiratory, with the most common symptoms including cough, fatigue, headache, muscle aches, and a loss of taste and/or smell, among others^2^. The symptoms that are centralized in the lungs present due to the damage to the alveolar tissue caused by the virus, specifically, the resulting pneumonia, which coincides with inflammation^3^. These symptoms are associated with the expression of angiotensin-converting enzyme 2 (ACE2) in the lungs, which is known as a receptor for SARS-CoV-2 and modulates the cellular entry of the virus. The spike (S) proteins of coronaviruses are known to bind to ACE2, and SARS-CoV-2 has been found to be more infectious than earlier coronaviruses since its binding affinity for ACE2 is higher^4^.

As the pandemic continues, increasing evidence has drawn attention to the various local and systemic inflammatory effects of the virus, such as cytokine storms^5^. These inflammatory effects allow the transition of focus from localized damage in the lungs to systemic damage in the body, specifically in the central nervous system (CNS), where the virus was shown to produce pathologies resembling various “classic” forms of neurodegeneration^6^. It has been noted that patients with dementia have an increased risk of contracting the virus, and it has also been suggested that those who contracted the virus had pathological symptoms that resembled those of neurodegenerative diseases such as Alzheimer’s disease (AD) and Parkinson’s disease (PD)^7–9^.

AD and PD are the most common neurodegenerative disorders, with ~6 and 1 million people, respectively, having the conditions in the United States alone in 2021, with both values expected to increase in the future as the average age increases^10,11^. In AD, the cognitive deficits are caused by abnormal accumulations of amyloid-β peptide (Aβ) and tau protein, called Aβ plaques and neurofibrillary tangles, respectively, which are considered the pathological hallmarks^12^. PD is a progressive neurological movement disorder^13^. Common symptoms of PD include tremor, rigidity, and bradykinesia, but nonmotor symptoms, such as depression and anxiety, also occur^13^. PD is characterized by the loss of dopaminergic neurons in the substantia nigra, and the pathological hallmarks are the abnormal deposition of misfolded proteins called Lewy bodies (LBs) and Lewy neurites (LNs), which are primarily composed of α-synuclein (α-syn)^13,14^.

Toll-like receptor 2 (TLR2) belongs to a family of pattern recognition receptors (PRRs) and is expressed on the surface of numerous cells, including innate immune cells^15^. TLR2 recognizes a variety of microbial components, such as lipopeptides and peptidoglycan^15^. While TLR2 plays an important role in the innate immune system, it has been demonstrated that TLR2 also plays an important role in the pathogenesis of neurodegenerative diseases, including AD and PD^16–20^. Therefore, TLR2 has been proposed as a new therapeutic target for these diseases^18,19,21^.

In addition to being a systemic respiratory disease, infection with SARS-CoV-2 induces neuropathology in patients^22–26^. Although recent studies have reported the presence of SARS-CoV-2 infection in the CNS^26,27^, it is still under debate whether SARS-CoV-2 can infect cells in the CNS^28^. Despite the controversy, accumulating evidence supports the invasion of SARS-CoV-2 into the CNS in patients, which could possibly induce further delayed neurological complications^28^. Therefore, it is reasonable to assume that there could be a receptor that might recognize a component of SARS-CoV-2 in the brain, such as TLR2. TLR2 has been known to interact with bacterial pathogens, and recent studies have demonstrated that TLR2 can also detect various viruses, including SARS-CoV-2^29–32^. In addition, TLR2 is widely expressed in brain resident cells, such as neurons and glial cells^33,34^. Therefore, in this review, we speculate that a pathogenic interaction between SARS-CoV-2 and TLR2 occurs in the CNS, and we will examine its potential effects on AD and PD pathology.

## Materials and methods

### Human brain immunohistochemical analysis

The paraffin-embedded brain sections of control and SARS-CoV-2-infected patients were kindly provided by Dr. Avindra Nath (National Institute of Neurological Disorders and Stroke). The procedure for immunochemical analysis has been described elsewhere^35,36^. Briefly, blinded brain sections were incubated with anti-ionized calcium-binding adapter molecule 1 (IBA-1, citrate buffer treatment, 1:2000, Wako Chemicals, Richmond, VA), anti-transmembrane protein 119 (TMEM119, citrate buffer treatment, 1:500, Abcam, Cambridge, UK), anti-cluster of differentiation 3 (CD3, citrate buffer treatment, 1:2000, Thermo Fisher Scientific, Waltham, MA), or anti-phospho-α-synuclein (S129) (81 A, citrate buffer treatment, 1:10,000, gift from Drs. Virginia Lee and John Trojanowski, University of Pennsylvania, PA) at 4 °C overnight. The next day, the sections were incubated with a biotinylated secondary antibody and detected with an avidin D-HRP detection system (ABC elite, Vector Laboratories, Burlingame, CA). The immunostained sections were imaged by an Olympus BX41 microscope (Olympus, Tokyo, Japan).

## SARS-CoV-2 and neurodegenerative disorders

### Neuropathology of SARS-CoV-2-infected patients

**Fig. 1:**
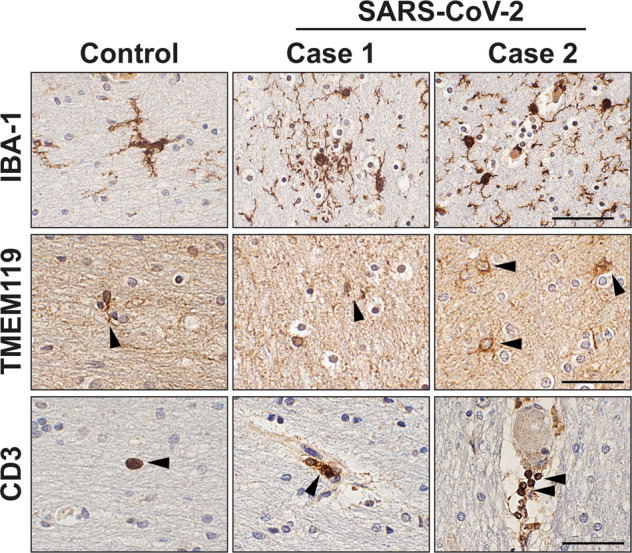
Representative immunohistochemical analysis of innate and adaptive immune cells in the frontal cortex of control and SARS-CoV-2-infected patients. White matter sections obtained from one control and two SARS-CoV-2-infected patients were immunostained with anti-IBA-1 and anti-TMEM119 for microglia and anti-CD3 for T cells. Scale bars represent 50 μm.

There is increasing evidence that patients infected with SARS-CoV-2 have neurological symptoms along with respiratory symptoms^37,38^. Approximately 36% of SARS-CoV-2-infected patients have neurological symptoms^39^. The common neurological symptoms of patients include headaches and nausea, but patients can also present with more severe neurological disorders, such as meningo-encephalitis and acute cerebrovascular disease^22–26^. Neuropathologies of SARS-CoV-2-infected patients are varied, but common neuroinflammatory findings have been reported, including astrogliosis, microgliosis, ischemia, hemorrhage, and microvascular lesions in the CNS of patients^40^. Similar to previous studies, our postmortem analysis revealed the activation of microglia in the patients’ brains (Fig. 1). Whether T cells infiltrate the CNS is controversial;^41,42^ however, a recent study suggested that subpopulations of CD3^+^ and CD4^+^ T cells infiltrate the CNS and interact with resident microglial cells in SARS-CoV-2-infected patients^43^. Our postmortem study also detected a small number of infiltrated CD3^+^ T cells in the cortex of SARS-CoV-2-infected patients (Fig. 1).

### How does SARS-CoV-2 enter the CNS?

**Fig. 2:**
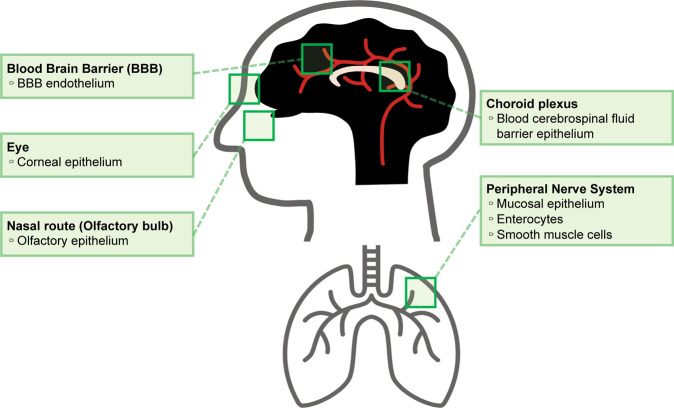
Potential entry routes for SARS-CoV-2 into the central nervous system (CNS). SARS-CoV-2 can infiltrate the CNS via the penetration of the blood-brain barrier (BBB), the blood-cerebrospinal fluid barrier in the epithelium of the choroid plexus, the corneal epithelium of the eye, the olfactory epithelium of the olfactory bulb (nasal route), and the peripheral nervous system, including the mucosal epithelium, enterocytes, and smooth muscle cells.

Although the expression of ACE2 is very limited in the CNS and the amount of SARS-CoV-2 present in the CNS after infection is still disputed^14,26^, postmortem studies have identified the existence of SARS-CoV-2 in the CNS of patients^44^. The presence of SARS-CoV-2 in the CNS was initially hypothesized due to anosmia that presented as a common symptom of the infection^45^. This led to the speculation of the olfactory bulb as a potential route of entry for the virus into the brain^46^. Meinhardt et al. suggested that the neural-mucosal interface could be a potential route for SARS-CoV-2 neuroinvasion (Fig. 2)^46^. However, the study also demonstrated the presence of the virus in other brain regions that had no direct connection to this interface, leading them to suggest the existence of other routes for SARS-CoV-2 neuroinvasion^46^. There are four other potential CNS entry mechanisms of SARS-CoV-2, although none have been proven (Fig. 2). Armocida and colleagues proposed that the virus could infect neurons in the peripheral nervous system and then take advantage of axonal transport to gain access to the CNS^47^. McQuaid et al. suggested the lateral ventricles and choroid plexus as a CNS entry mechanism for SARS-CoV-2^48^. Since these regions contain epithelial cells, which express ACE2, it has been suggested that the virus could cross the blood-cerebrospinal fluid barrier and enter the choroid plexus and ventricular system. Recent studies demonstrated that the expression of ACE2 is relatively high in the corneal epithelium and suggested that ocular conjunctival inoculation was enough to cause COVID-19^49,50^. In addition, various sampling studies identified the presence of SARS-CoV-2 RNA within regions of the visual system, such as the retina, optic nerve, conjunctiva, and vitreous body, in patients with confirmed SARS-CoV-2 diagnoses^51,52^. A recent study revealed that the S protein of the novel coronavirus can cross the blood-brain barrier (BBB)^53^. Therefore, it has also been suggested that SARS-CoV-2 could impair the functional integrity of the BBB^54,55^.

### Alzheimer’s disease and SARS-CoV-2

A recent clinical study found that the risk of SARS-CoV-2 infection for patients with dementia was increased 2–3-fold compared with cognitively healthy individuals^8^. In addition, the levels of total tau, phosphorylated tau181, and glial fibrillary acidic protein, all biomarkers for AD, were elevated in SARS-CoV-2-infected patients with severe symptoms, suggesting a potential correlation between AD and SARS-CoV-2 infection severity^56^. Transcriptomic and interactomic data also showed a relationship between SARS-CoV-2 and β-amyloid production and clearance, leading to the conclusion that SARS-CoV-2 infection may exacerbate AD neuropathology^57^. In addition, patients with the homozygous allele apolipoprotein E4 (APOE4), an AD-associated gene, showed an increased risk for SARS-CoV-2 infection, and APOE4 may also affect the severity of the host response to infection^58,59^. Furthermore, it was found that SARS-CoV-2-infected patients with AD had a higher rate of death due to the disease than SARS-CoV-2-infected patients without AD^60^.

In addition to viral infection, the characteristic behaviors of AD patients may increase the risk for SARS-CoV-2 infection and severity. First, patients may not be able to follow the recommendations from public health providers to reduce the spread of the virus^61^. Second, the lack of social interaction due to the pandemic may increase mental and psychological stress in AD patients^62^. Increased psychological stress further accelerates the deterioration of cognitive function in AD patients^62^.

ACE2 is a known receptor for SARS-CoV-2, and its role in AD has been extensively studied during the SARS-CoV-2 pandemic; however, its role in AD is controversial^63^. A recent study found an inverse correlation between ACE2 activity and AD patient neuropathology, such as the accumulation of Aβ and phosphorylated tau^64^. In addition, Kehoe and colleagues reported a reduction in ACE2 activity in the brain homogenate of AD patients carrying the APOE4 allele^64^. However, Lim and colleagues showed an increased level of ACE2 in the brain tissue of AD patients^65^. Zhao and colleagues found that ACE2 expression was upregulated in the occipital and temporal lobes and the hippocampal CA1 region in AD patients compared to healthy controls^66^. Therefore, further studies are required to evaluate the role of ACE2 in AD pathogenesis.

### Parkinson’s disease and SARS-CoV-2

To date, the risk factor associating PD with SARS-CoV-2 infection has not been clearly identified^67^. However, it has been suggested that SARS-CoV-2 infection may trigger parkinsonism symptoms in healthy individuals^68^. A case study by Lee and colleagues suggested the potential effects of SARS-CoV-2 infection on the dopaminergic mechanisms that led to the development of dysphagia in PD patients^69^. In addition, a recent study reported that infection with SARS-CoV-2 may worsen the symptoms of PD^70^. It was demonstrated that the nucleocapsid (N) protein of SARS-CoV-2 sped up the process of α-syn aggregation in vitro^71^.

The SARS-CoV-2 pandemic has also increased the level of psychological stress in PD patients^72^ which may worsen the symptoms of PD. For example, the accumulation of psychological stress has been shown to cause the temporary aggravation of motor symptoms^73,74^. One observational study found that 40% of PD patients showed an exacerbation of their motor symptoms during the pandemic^75^. In addition, the limitation of physical exercise increases psychological stress^72^.

## TLR2 as a potential SARS-CoV-2 receptor in the CNS

### TLR2

Toll-like receptors (TLRs) belong to a family of innate immune receptors known as PRRs^15^. To date, 10 human (TLR1-10) and 13 murine (1-13) subtypes of TLRs have been identified^76^. TLRs are type I transmembrane proteins and have a leucine-rich repeat (LRR) motif, transmembrane domain, and cytoplasmic Toll/IL-1 receptor (TIR) domain^77^. TLRs are abundantly expressed in multiple peripheral organs but are also expressed in the neuronal and nonneuronal cells of the CNS^78^. TLRs can recognize both exogenous pathogen-associated molecular patterns (PAMPs) and endogenous damage-associated molecular patterns (DAMPs)^79^. TLRs form a homo-/heterodimer to recognize different shapes of pathogens^80^. For example, the TLR4 homodimer recognizes the lipopolysaccharide of gram-negative bacteria, and the TLR3 homodimer recognizes double-stranded RNA^81^. Once activated, TLRs trigger intracellular signaling cascades via myeloid differentiation primary-response protein 88 (MyD88), except for TLR3, which initiates signaling via Toll/interleukin 1 receptor domain-containing adaptor interferon-β (TRIF), thereby resulting in the induction of inflammatory cytokines and chemokines^15^.

### The role of TLR2 in AD

TLR2 is an innate immune receptor, but increasing evidence demonstrates its role in neurodegenerative diseases, including AD and PD^18,82^. Recent genetic studies identified TLR2 as a risk factor for late-onset AD (LOAD) in Han Chinese and Azeri Turk ancestry populations^83,84^. While genetic association studies of TLR2 with AD are still open to debate^76^, accumulating in vitro and in vivo studies provide evidence for the pathogenic role of TLR2 in AD.

In microglia, the brain resident innate immune cells, TLR2 mediates fibrillar Aβ-induced immune responses^85^. In addition, the activation of TLR2 enhances pathogenic Aβ uptake in microglia^86^. On the other hand, genetic depletion of TLR2 reduces the Aβ42-induced immune response and enhances Aβ clearance in cultured microglia^16,82^. In an animal model of AD (APP/PS1 mice), functional inhibition of TLR2 decreases microgliosis, astrogliosis, Aβ plaque deposition, and phosphorylated tau accumulation in the brain regions, thereby improving cognitive function^19,87,88^. In addition, genetic depletion of TLR2 shows protective effects against memory and cognitive impairments in an AD mouse model^89,90^. The expression of TLR2 is increased in AD patients and animal models^91,92^. Furthermore, immunohistochemical analysis demonstrates that the localization of microglial TLR2 is associated with Aβ plaques in the brains of AD patients and aged mouse models^82,85,93^.

### TLR2 in PD

Although the pathogenic role of TLR2 in PD was demonstrated a few years later than that in AD, it has also been extensively studied for this short period^18^. Genetic associations of TLR2 polymorphisms with PD were identified in northeastern Han Chinese and Greek populations^94,95^. In 2013, we first demonstrated that neuron-released oligomeric forms of α-syn activated microglial TLR2, thereby inducing neurotoxic inflammation through the activation of nuclear factor kappa B (NF-κB)^17,96^. This finding was supported by subsequent in vitro and in vivo studies. Roodveldt et al. demonstrated that pretreatment with a TLR2 agonist, but not other TLR agonists, increased microglial susceptibility against α-syn^97^. Qiao et al. showed that functional and genetic inhibition of TLR2 prevented microglial responses against neuron-released α-syn^98^. Daniele et al. reported that α-syn treatment induced microglial inflammatory responses by forming a TLR1/2 heterodimer complex^99^. We also demonstrated that leucine-rich repeat kinase 2 (LRRK2), a PD-associated gene, and nuclear factor of activated T cell 1 (NFAT1) are downstream signaling molecules of TLR2 in microglia, thereby modulating neurotoxic microglial activation^100^. In a mouse model of PD, Drouin-Ouellet et al. reported that the overexpression of α-syn increased the expression of TLR2^101^. La Viola et al. demonstrated that oligomeric forms of α-syn induced memory impairment through TLR2^102^. Interestingly, exercise showed a protective effect in a 1-methyl-4-phenyl-1,2,3,6-tetrahydropyridine (MPTP)-induced PD mouse model via the downregulation of TLR2 and downstream signaling molecules, including MyD88^103^. In PD patients, the level of TLR2 was elevated in the blood compared to healthy controls^101^. Furthermore, the expression of TLR2 was also increased in the specific brain regions of PD patients and aged animal models in accordance with disease stages^91,101^.

Although TLR2 is primarily expressed in innate immune cells, recent studies have demonstrated that neurons also express TLR2^81,104^, which has been demonstrated to play an important role in PD. We demonstrated that the activation of neuronal TLR2 impaired autophagy through the AKT/mammalian target of rapamycin signaling cascade, thereby inducing the accumulation of neurotoxic α-syn aggregates^105^. These findings were supported by Dzamko and colleagues, who also demonstrated the elevation of neuronal TLR2 in the brains of PD patients^106^.

The neuron-to-neuron and neuron-to-glial transmission of α-syn has been proposed to play a central role in PD pathogenesis and disease progression^107^. Although the primary role of TLR2 is recognizing pathogens, TLR2 also modulates pathogen phagocytosis in cells^108^. Genetic or pharmacological activation of TLR2 increased extracellular α-syn uptake by neuronal and glial cells, while it was inhibited by genetic and functional depletion of TLR2^17,21,105^. Specifically, the α-syn transmission assay indicated that the activation of TLR2 not only increased α-syn transmission in neurons but also increased its propagation^21^. In microglia, the internalization of monomeric α-syn was not affected by TLR2, but the uptake of α-syn oligomer was significantly decreased by TLR2 inhibition in the cells^17^. In addition, the deposition of α-syn increased in astrocytes that did not express α-syn in either PD patients or mouse models^21^. These observations were reproduced in PD models in which functional inhibition of TLR2 significantly reduced astroglial α-syn deposition in both a PD mouse model and a neuron-to-astrocyte α-syn monitoring system^21^.

Given that TLR2 plays an important role in PD, targeting TLR2 has been proposed as a promising immunotherapeutic option for the disease^18^. Indeed, the administration of a TLR2 functional blocking antibody improved α-syn neuropathology, neuroinflammation, and motor behavioral deficits in a PD mouse model^21^.

### TLR2 and SARS-CoV-2

Lipopeptides and gram-positive bacteria-derived lipoprotein are considered the traditional ligands of TLR2^15^. However, increasing evidence also supports the interaction of TLR2 with viruses. Varicella-zoster virus activates the inflammatory response in monocytes via TLR2^109^. Furthermore, various viral proteins, such as the structural proteins (p17, p24, and gp41) of human immunodeficiency virus 1, the core protein of hepatitis C virus, and glycoprotein B of herpes simplex virus 1, have been known to interact with TLR2, thereby inducing cytokine gene expression^110^. In addition, it was found that the S protein of SARS-CoV-1 activated TLR2 in peripheral leukocytes, which resulted in the induction of proinflammatory cytokine and chemokine gene expression, including interleukin-6 (IL-6) and TNF-α^111^.

The exact pathogenic mechanism of SARS-CoV-2 is still largely unknown. However, increasing evidence supports that TLRs might play a role during SARS-CoV-2 pathogenesis^112^. It has been shown that the surface proteins of SARS-CoV-2 could behave as a PAMP, thereby inducing the upregulation of inflammatory factors in the rodent model through TLR2 and TLR4^113^. Prophylactic administration of a TLR2 agonist showed a protective effect against SARS-CoV-2 infection and decreased virus transmission through the activation of the innate immune system^114^. More importantly, Zheng et al. demonstrated that TLR2 can sense the envelope (E) protein of SARS-CoV-2, thereby inducing an inflammatory response^30^. Signaling molecules downstream from TLR2, including MyD88 and TRIF, were significantly increased in SARS-CoV-2-infected patients with severe/critical conditions compared to healthy controls. In addition, a pharmacological inhibitor of TLR2 inhibited the inflammatory responses of human leukocytes against SARS-CoV-2 infection. Furthermore, genetic depletion of TLR2 prevented inflammation and tissue damage in the lungs of mice expressing human ACE2. These findings suggest that the surface proteins of SARS-CoV-2 induce the activation of TLR2, leading to inflammatory responses.

## Prospective: Does SARS-CoV-2 affect AD and PD patients through TLR2?

**Fig. 3:**
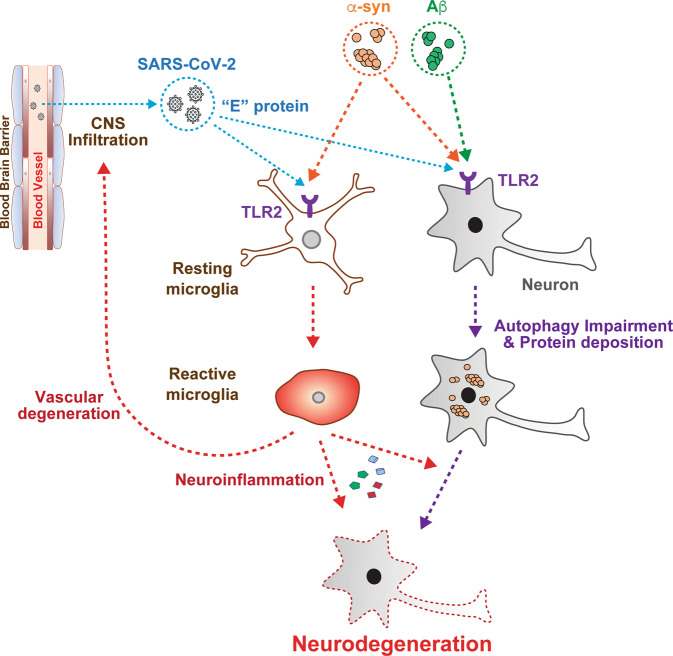
Potential interaction mechanism of SARS-CoV-2 and TLR2 in AD and PD. E protein of brain-infiltrated SARS-CoV-2 induces microglial TLR2, thereby increasing the susceptibility of TLR2 to α-syn and Aβ oligomers. The activation of microglial TLR2 by the E protein of SARS-CoV-2, α-syn, or Aβ increases the release of neurotoxic, proinflammatory cytokines, which may also induce vascular degeneration in the brain, thereby increasing SARS-CoV-2 infiltration into the CNS. In neurons, the E protein of SARS-CoV-2 may activate neuronal TLR2, which impairs neuronal autophagic processes, resulting in an accumulation of neurotoxic α-syn aggregates.

ACE2 is a primary receptor for SARS-CoV-2. After infection, the number of SARS-CoV-2-positive cells rapidly increases in the host peripheral system^115^. However, the viral load in the CNS is lower than that in the periphery^26^. Based on these observations, we speculate that SARS-CoV-2 infection is limited in the CNS during the early infection period for two reasons: the inhibition of SARS-CoV-2 CNS infiltration by the existence of a physical barrier (the BBB) and the low level of SARS-CoV-2 high-affinity receptors in the CNS^116^. However, persistent infection with SARS-CoV-2 leads to excessive peripheral immune responses, which could induce damage to the BBB^117^. Therefore, a greater number of viruses can infiltrate the CNS via the damaged barriers, which may increase the chance that a viral component will meet a responding receptor, such as TLR2, in the CNS (Fig. 3).

**Fig. 4:**
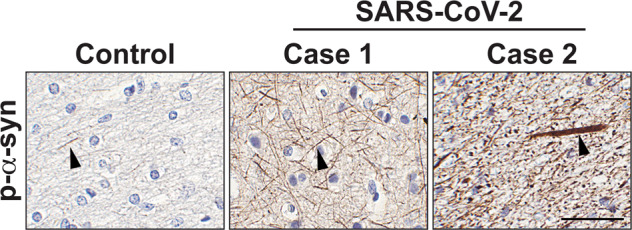
Representative immunohistochemical analysis of phosphorylated-α-syn in the frontal cortex of control and SARS-CoV-2-infected patients. White matter sections obtained from one control and two SARS-CoV-2-infected patients were immunostained with anti-phospho-α-syn. The arrowhead represents the LN-like accumulation of phospho-α-syn (p-S129). Scale bar represents 50 μm.

Many of the severe and critical conditions of SARS-CoV-2-infected patients result in death^115^. However, vaccination against SARS-CoV-2 will help reduce the numbers of patients who become severely or critically ill. In addition, developing a medication for COVID-19 would reduce the death rate of patients in the future. To date, two treatments have been approved by the FDA: an antibody cocktail targeting SARS-CoV-2 (REGN-COV2, REGENERON) and an oral antiviral medicine (Molnupiravir, Merck). On the other hand, CNS-infiltrated viruses might survive longer than those in the peripheral system due to the lack of an adaptive immune system and the high selectivity of the BBB against drugs^117^. Therefore, this prolonged presence of SARS-CoV-2 in the CNS may cause further problems in the brain that might not present until much later. Specifically, SARS-CoV-2 viral components may directly affect patients with neurodegenerative diseases. According to Zheng et al., the E protein of SARS-CoV-2 is activated and induces TLR2 expression in innate immune cells^30^. Microglia, brain resident innate immune cells, express TLR2, which plays a critical role in the neuroinflammation of AD and PD patients^17,85^. Therefore, we speculate that the viral components, especially the E protein, of brain-infiltrated SARS-CoV-2 induces the activation of microglial TLR2, thereby increasing the susceptibility of TLR2 to Aβ and α-syn deposition in patients (Fig. 3). The chronic activation of TLR2 can induce chronic neuroinflammation, which will accelerate disease pathogenesis^118^. TLR2 is also expressed in neurons, and prolonged infection with SARS-CoV-2 may induce neuronal TLR2 activation in the brain. The induction of neuronal TLR2 is associated with pathological α-syn neuron-to-neuron transmission and propagation, which is known to be associated with disease progression^18^. In addition, the activation of neuronal TLR2 has been shown to impair neuronal autophagy, thereby increasing abnormal accumulation of neurotoxic misfolded proteins, such as α-syn^105^. Therefore, the induction of neuronal TLR2 susceptibility by the E protein of SARS-CoV-2 may lead to the deposition of abnormal protein in the cells, thereby affecting the disease onset and/or accelerating the disease progression of proteinopathy-associated neurodegenerative diseases. Indeed, our preliminary postmortem analysis revealed that the accumulation of phosphorylated α-syn, one of the pathogenic forms of α-syn, was increased in the brains of SARS-CoV-2-infected patients (Fig. 4). For these reasons, further detailed studies are required to understand the pathogenic interaction between SARS-CoV-2 and TLR2 and the potential of TLR2 as target for COVID-19 treatment.
